# Measuring the Overall Burden of Early Childhood Malnutrition in Ghana: A Comparison of Estimates From Multiple Data Sources

**DOI:** 10.34172/ijhpm.2020.253

**Published:** 2020-12-22

**Authors:** John Paul Kuwornu, Jonathan Amoyaw, Taru Manyanga, Elizabeth J. Cooper, Elvis Donkoh, Amos Nkrumah

**Affiliations:** ^1^Research Department, Saskatchewan Health Authority, Regina, SK, Canada.; ^2^Department of Sociology, University of Saskatchewan, Saskatoon, SK, Canada.; ^3^School of Epidemiology and Public Health, Faculty of Medicine, University of Ottawa, Ottawa, ON, Canada.; ^4^Faculty of Kinesiology and Health Studies, University of Regina, Regina, SK, Canada.; ^5^Department of Mathematics and Statistics, University of Energy and Natural Resources, Sunyani, Ghana.; ^6^Department of Sociology & Anthropology, Mount Saint Vincent University, Halifax, NS, Canada.

**Keywords:** Under Nutrition, Sustainable Development Goals, Demographic Health Survey, Prevalence of Malnutrition, Ghana

## Abstract

**Background:** Childhood malnutrition contributes to nearly half (45%) of all deaths among children under 5 globally. The United Nations’ Sustainable Development Goals (SDGs) aims to end all forms of malnutrition by 2030; however, measuring progress towards these goals is challenging, particularly in countries with emerging economies where nationally-representative data are limited. The primary objective of this study was to estimate the overall burden of childhood malnutrition in Ghana at national and regional levels using 3 data sources.

**Methods:** Using data from the long-standing Ghana Demographic and Health Surveys (GDHS), Ghana Multiple Indicator Cluster Survey (GMICS), and the emerging Ghana Socioeconomic Panel Survey (GSPS), we compared the prevalence of malnutrition using the extended composite index of anthropometric failure (eCIAF) for the period 2008- 2011. This study included data for children aged 6-59 months and calculated all anthropometric z-scores based on the World Health Organization (WHO) Growth Standards. We tested for differences in malnutrition subtypes using two-group configural frequency analysis (CFA).

**Results:** Of the 10 281 children (6532 from GMICS, 2141 from GDHS and 1608 from GSPS) included in the study, the only demographic difference observed was the children included in the GSPS were slightly older than those included in the GDHS and GMICS (median age of 36 vs 30 vs 33 months, *P*<.001). Based on the eCIAF, the overall prevalence of malnutrition at the national level was higher among children in the GSPS (57.3%, 95% CI: 53.9%–60.6%), followed by the GDHS (39.7%, 95% CI: 37.0%–42.5%), and then those in the GMICS (31.2%, 95% CI: 29.3%–33.1%). The two-group CFA showed that the 3 data sources also estimated different prevalence rates for most of the malnutrition subtypes included in the eCIAF.

**Conclusion:** Depending on the data source adopted, our estimates of eCIAF showed that between one-third and half of all Ghanaian children aged 6-59 months had at least one form of malnutrition over the period 2008-2011. These eCIAF estimates should complement the commonly reported measures such as stunting and wasting when interpreting the severity of malnutrition in the country to inform policy decisions.

## Background

**Key Messages**

** Implications for policy makers**

- The conventional measures of early childhood malnutrition, such as stunting and wasting show that Ghana is making good progress in meeting the United Nations’ Sustainable Development Goals (SDGs) for malnutrition by 2030.
- However, the extended composite index of anthropometric failure (eCIAF), a broader alternative to the conventional measures of malnutrition, shows that the country still has very high prevalence rates of early childhood malnutrition.
- By using multiple nationally-representative data sources, our study identified regions with the highest malnutrition prevalence rates to be prioritized for reducing early childhood malnutrition.

** Implications for the public**

Children with multiple anthropometric failures are more likely to experience ill-health and face a higher mortality risk, and should be prioritized for interventions. Our study identifies high risk children who suffer from multiple subtypes of malnutrition for targeted interventions.

Early childhood malnutrition has enduring negative socioeconomic and health consequences into late adulthood,^1,2^ continues to be a major determinant of child survival in countries with emerging economies,^3^ and contributes to almost half (45%) of all deaths among children under the age of 5 years globally.^4^ For the children who survive, early childhood malnutrition increases the risk of developing health conditions such as diabetes and cardiovascular diseases, impaired cognitive development, reduced school achievement, and lower economic productivity in adulthood.^5,6^

 Early childhood malnutrition comprises both over- and under-nutrition, 2 pandemics which seriously threaten human health^7^ and are deficiency diseases caused by poor nutrition.^8^ Early childhood under-nutrition may manifest in stunting (ie, low height-for-age [HAZ]), wasting (ie, low weight-for-height [WHZ]), underweight (ie, low weight-for-age [WAZ]), or their combinations,^7^ while over-nutrition may manifest in overweight or obesity. Current worldwide estimates indicate that in 2018, significant numbers of children under the age of 5 years were affected by stunting (149 million), wasting (49 million), and overweight (40 million), with Asia and Africa continuing to bear the greatest share of the global burden of early childhood malnutrition.^9^

 Compared to other African countries, Ghana has made significant strides in reducing childhood malnutrition. According to the 2015 final report^10^ on Ghana’s performance on the Millennium Development Goals (MDGs), between 1993 and 2014, the country had successfully cut in half the proportion of children who were underweight and presented with wasting, as targeted in the MDGs, but failed to meet the target for stunting (actual of 19% against a 2014 target of 7%). In spite of the progress at the national level, there have been significant inequalities across the country. Particularly, the prevalence of stunting among children under 5 is alarming in rural and economically deprived areas.^11^ Although the conventional measures of malnutrition (ie, wasting, underweight, overweight, and stunting) reflect distinct biological processes, none of them are designed to give an overall estimate of malnutrition in a given population due to the overlapping nature of these measures.^12,13^ To enable an overall estimate of undernutrition in a population, Svedberg^14^ and Nandy et al^15^ developed the composite index of anthropometric failure (CIAF); which has further been expanded by other authors^16,17^ to account for over-nutrition and called extended composite index of anthropometric failure (eCIAF). Compared to the conventional measures, the CIAF and eCIAF are better in guiding policy interventions because they allow for distinguishing children who are experiencing single versus multiple anthropometric failures. Children with multiple anthropometric failures are more likely to experience ill-health and face a higher mortality risk,^14,18^ thus, should be prioritized for interventions. Regardless of the improvements made as indicated by the conventional measures of malnutrition, early childhood malnutrition remains a significant public health issue in Ghana and tracking the overall estimate of malnutrition in the population using indices such as the CIAF and eCIAF would be invaluable for developing evidence-informed policy interventions.

 One of the main challenges of meeting the targets set for early childhood malnutrition in the Sustainable Development Goals (SDGs) is the lack of comprehensive and consistent data to enable tracking of progress.^19^ For example, SDG 2.2 targets an end to all forms of malnutrition by 2030, including achieving the internationally agreed targets on stunting (40% reduction) wasting (less than 5% prevalence), and overweight (less than 6% prevalence) in children under 5 years of age by 2025.^3,20^ However, the reliable data sources needed to track progress towards achieving these specific goals of early childhood malnutrition in sub-Saharan Africa are scarce.^21^ In Ghana for instance, 2 nationally representative cross-sectional household surveys, the Ghana Demographic and Health Surveys (GDHS) and the Ghana Multiple Indicator Cluster Survey (GMICS) have been the major data sources for tracking child and maternal health indicators, since the 1980s and 1990s, respectively. In 2009, the Ghana Socioeconomic Panel Survey (GSPS) was launched, with the first wave of data collection concluding in 2010. The GSPS was designed to provide a scientific framework for tracking and studying changes in the country’s developmental process.^22^

 The GDHS, GMICS and GSPS use similar survey methodology and have significant overlap in the range of indicators they cover, including education, health, livelihood, and social protection. Like other countries aiming to achieve SDG 2.2, Ghana could benefit from consistently using these nationally representative data sources to track progress. However, important reports such as the 2015 report^10^ on Ghana’s performance on childhood malnutrition indicators in the MDGs only relied on data from the GDHS. Given that the GDHS, GMICS, and GSPS are conducted on different time schedules, the 3 data sources could provide complementary and therefore comprehensive insights on the burden of early childhood malnutrition in the country. For example, GDHS alone can only track childhood malnutrition in 5-year intervals, whereas the combination of the GDHS and GMICS reduces this interval to 3 years; since each survey is conducted in the intervening period of the other. Clearly, monitoring the progress towards SDG 2.2 using the combination of these 3 data sources provides information to policy-makers within shorter time intervals and allows for timely implementation of interventions. Studies that provide comparisons of baseline estimates of early childhood malnutrition across these data sources can encourage the triangulation of the 3 data sources for monitoring childhood malnutrition in Ghana.

 Previous studies on childhood malnutrition in Ghana have examined the correlates of malnutrition in a nationally representative sample,^23^ assessed the factors affecting the uptake of intervention programs to prevent malnutrition,^24^ or focused on only deprived areas such as the Northern region of Ghana.^25^ To the best of our knowledge, estimates of the overall burden of childhood malnutrition at both national and regional levels have not been previously reported in Ghana. Therefore, the primary objective of this study was to use the CIAF and eCIAF indices to estimate the overall prevalence of early childhood malnutrition in Ghana at both national and regional levels, and compare these estimates across the GMICS, GDHS, and GSPS data sources. A secondary goal of the study was to compare the magnitude of change in the conventional measures of malnutrition over 2 survey cycles for each of the data sources; an analysis useful for providing further context to the country’s progress towards achieving SDG 2.2. This study contributes to the literature by measuring the overall burden of malnutrition not only at the national level but also at the regional levels to capture spatial variations in malnutrition across the country. Insights from this paper will be useful for targeted policy initiatives, especially in high-risk areas, to reduce the prevalence of childhood malnutrition and improve child health in general.

## Methods

###  Data Sources and Study Sample

 This study relied on data obtained from 3 nationally representative surveys in Ghana, namely the 2008 GDHS, the 2009-2010 GSPS, and the 2011 GMICS. The 3 surveys were selected because they were the closest in years to one another between the 3 data sources (Table 1). Secondly, they were selected because they represent a meaningful baseline for tracking progress towards SDG 2.2. As part of efforts to meet the SDGs, the World Health Assembly Resolution 65.6 endorsed a comprehensive implementation plan on maternal, infant and young child nutrition in 2012, which specified a set of 6 global nutrition targets that should be met by 2025. The 2008 GDHS, 2009-2010 GSPS and 2011 GMICS data used in this study are likely to have been the most updated data sources on childhood malnutrition in Ghana at the time these targets were adopted, and can serve as useful baselines for ascertaining progress. The 2008 GDHS was carried out by the Ghana Statistical Service and the Ghana Health Service, with technical assistance from MEASURE DHS program.^26^ The 2009-2010 GSPS was conducted and managed by the Economic Growth Center at Yale University and the Institute of Statistical, Social, and Economic Research at the University of Ghana.^27^ The 2011 GMICS was carried out by the Ghana Statistical Service, with technical support from the United Nations Children’s Fund (UNICEF) and other international organizations.^28^

**Table 1:** Data Availability of the Three Nationally Representative Surveys Since 1990s

**Data Source**	**Years Survey Was Conducted**				
GMICS	1995	2006	2011	2017-2018^a^	
GDHS	1993	1998	2003	2008	2014
GSPS	2009-2010	2013-2014^a^	2017-2018^a^		

Abbreviations: GMICS, Ghana Multiple Indicator Cluster Survey; GDHS, Ghana Demographic Health Survey; GSPS, Ghana Socioeconomic Panel Survey. ^a^Data not publicly available at the time this study was conducted.

 We used the children sub-sample from each of the data sources, focusing on those aged 6-59 months who had their anthropometric (ie, weight and height) measurements recorded. Similar to previous studies,^29,30^ we excluded children aged less than 6 months because of the possibility of a significant decrease in adiposity during this period. We also excluded children based on the World Health Organization (WHO) flagging criteria^31,32^ for unusual or biologically implausible anthropometric measurements, as these height and/or weight values and their z-score derivatives are usually considered to be errors in measurement and/or recording.

###  Study Procedure and Data Collection

 The 2008 GDHS, 2009-2010 GSPS, and 2011 GMICS all used a 2-stage stratified sample design. At the first stage, 411, 334, and 810 clusters were selected for the 2008 GDHS, 2009-2010 GSPS, and 2011 GMICS, respectively. These clusters were selected using a simple random sampling technique from an updated master sampling frame constructed from the 2000 Ghana Population and Housing Census. For the second stage, 30 households were selected from each cluster selected at the first stage for the 2008 GDHS whereas 15 households were selected for both the 2009-2010 GSPS and 2011 GMICS. In total, 11 913 occupied households were selected in the 2008 GDHS, out of which 11 778 were successfully interviewed, yielding a household response rate of about 99%.^26^ For the 2009-2010 GSPS, 5041 occupied households selected and 5009 were successfully interviewed, resulting in a household response rate of over 99%.^22^ The household response rate was close to 100% in the 2011 GMICS, given that 11 925 households were successfully interviewed out of a total of 11 970 occupied households selected.^28^ The 3 surveys conducted face-to-face interviews with all participants.

###  Data Access and Ethics Statement

 The GDHS requires an authorization from the custodians before the data can be accessed. The authorization for access and access to the whole DHS database can be completed through the DHS program website at the address https://dhsprogram.com/. The 2009-2010 GSPS data is publicly available, but its usage is subject to terms and conditions of the data custodians. The data can be accessed from The World Bank Microdata Library website at the address https://microdata.worldbank.org/index.php/catalog/2534. Similar to the GDHS, the GMICS requires an authorization from the data custodians before the data can be accessed. The authorization for access and access to the whole MICS database can be completed through the MICS program website at the address https://mics.unicef.org/surveys. The 3 surveys fulfilled all ethical requirements and participants’ records in the databases were anonymized, fully concealing the identities of all participants. No further approvals are required for the retrospective use of these data.

###  Study Variables and Measurements

 The study variables included age (in months), sex (male vs female), region of residence (Ashanti, Brong Ahafo, Central, Eastern, Greater Accra, Northern, Volta, Western, Upper East, and Upper West), height (in cm) and weight (in kg). Except for region of residence, the other variables were used to calculate a range of z-scores, including HAZ, WAZ, WHZ, and BMI-for-age (BMIZ) using the 2006 WHO growth standards.^33^ The following standard case-definitions were applied to each record to classify children as: wasted: WHZ <-2.0, stunted: HAZ <-2.0, underweight: WAZ <-2.0, or overweight: WAZ >2.0. We also used the CIAF and eCIAF to measure the overall prevalence of malnutrition (ie, over- and under-nutrition). The CIAF identifies 7 mutually exclusive subtypes of undernutrition, including: (*a*) no failure: normal WAZ, HAZ, and WHZ, (*b*) wasting only: WAZ <-2.0 but normal HAZ and WHZ, (*c*) wasting and underweight: WAZ and WHZ <-2.0 but normal HAZ, (*d*) stunting, wasting, and underweight: HAZ, WAZ, and WHZ <-2.0, (*e*) stunting and underweight: HAZ and WHZ <-2.0 but normal WAZ, (*f*) stunting only: HAZ <-2.0 but normal WAZ and WHZ, and (*y*) underweight only: WHZ <-2.0 but normal HAZ and WAZ. The CIAF sums all the subtypes (*b*) to (*y*) of undernutrition to estimate the overall prevalence of early childhood undernutrition in a population. The eCIAF accounts for over-nutrition by adding 2 more groups to the CIAF, namely (*g*) stunting and overweight, and (*h*) overweight only. Thus, the eCIAF can estimate the overall prevalence of early childhood malnutrition in a population.

###  Data Analysis

 We compared the distributions of sex, age, height, weight, HAZ, WAZ, WHZ, BMIZ, stunting, wasting, underweight, and overweight between the 3 data sources; using the chi-square test or analysis of variance, as appropriate.

 Further, we used quantile regression models to make sex- and age-specific comparisons of the entire distributions of HAZ, WAZ, WHZ, and BMIZ between the GDHS, GMICS, and GSPS data sources. Quantile regression allows the estimation and inference on quantiles of a distribution without assumptions of normality and equality of variance required for standard regressions on mean values.^34^ Similar to Seirs et al,^35^ we generated quantile regression fits for every 2.5th percentile from the 0.05th to 0.95th quantiles of the 4 z-score values for each sex and age group. Estimates for each quantile were connected with lines and polygons were used to represent the 95% confidence intervals for the estimates, with non-overlapping confidence polygons indicating statistically significant differences.

 We used the eCIAF to calculate and plot the overall prevalence of early childhood malnutrition for each of the 10 regions in the country. These prevalence estimates were weighted to be regionally representative and reflect the regional variations in malnutrition across the country. We applied the appropriate weights (ie, PERWEIGHT for the GDHS, hhweight3 for the GSPS, and chweight for the GMICS) for each of the surveys. Given the 2-stage stratified sample design of the survey, we included information on both the primary and secondary sampling units, as well as using the region of residence as strata in specifying the survey design effects in the function *svydesign* of the R *survey* package.

 We used 2 methods to test for differences between the GDHS, GMICS, and GSPS for each of the subtypes of malnutrition captured in the CIAF and eCIAF. First, we calculated the prevalence of each of the subtypes of malnutrition in the eCIAF with their 95% confidence intervals, with non-overlapping confidence intervals indicating statistically significant differences. Similar to the weighting procedure explained above, these prevalence estimates were weighted to be nationally representative. Second, we used a two-group configural frequency analysis (CFA) to identify the subtypes of CIAF that are significantly different between the GDHS, GMICS, and GSPS. Unlike using confidence intervals to ascertain statistical significance, the CFA is a nonparametric method useful for analyzing the frequencies in multi-way contingency tables.^36^ In our two-group CFA, the observed frequencies were compared with expected frequencies in order to identify the “discrimination types” (ie, subtypes of undernutrition which are significantly different between each combination of 2 data sources). We made comparisons between the GSPS and GDHS, the GSPS and GMICS, and the GDHS and GMICS.

 With regards to the secondary objective of the study, we compared the changes in the prevalence of stunting, wasting, and overweight across the data sources. For the GDHS, we compared our estimates from the 2008 survey to the latest prevalence estimates from the 2014 survey.^37^ For the GMICS, we compared our estimates from the 2011 survey to the latest prevalence estimates from the 2017-2018 survey.^28^ Although the second wave of data collection for the GSPS took place in 2014-2015, the data are currently not publicly available, thus we were unable to report on the changes in prevalence estimates from this data source.

 For all tests conducted in this study, statistical significance was set at 0.05 alpha level. All data restructuring and analyses were conducted in R version 3.6.0.^38^ Specifically, we used the R-packages *data.table*^39^ for data preparation, *confreq*^40^ for the CFA, *quantreg*^41^ for the quantile regression, *ggmap*^42^ for mapping regional malnutrition prevalence rates, *survey*^43^ for weighting prevalence estimates to be nationally representative, *zscorer*^44^ for calculating anthropometric z-scores in the GSPS data, and *ggplot2*^45^ for plotting the anthropometric z-scores.

## Results

 After applying the exclusion criteria (ie, WHO flagging rules = 300 and children <6 months old = 718), a total of 6532 children were included in the GMICS. Using the same exclusion criteria (ie, WHO flagging rules = 306 and children <6 months old = 238), a total of 2141 children were included in the GDHS. Similarly, applying the same exclusion criteria (ie, WHO flagging rules = 733 and children <6 months old = 74) yielded a total of 1608 children to be included in the GSPS. Table 2 presents the demographic and anthropometric characteristics of the children recorded in each of the 3 data sources. The proportion of girls is similar in all data sources (49.2 in the GMICS vs 50.0% in the GDHS vs 50.1% in the GSPS). However, the children included in the GSPS were approximately 6 months older than those included in the GDHS and about 3 months older than those included in the GMICS (median age of 36 vs 30 vs 33 months, *P* < .001). All the anthropometric measures were marginally higher in the GSPS compared to the GMICS and GDHS. Furthermore, except for underweight, the prevalence estimates were higher in the GSPS followed by the GDHS and then the GMICS for stunting (35.4% vs 30.0% vs 29.1%, *P* < .001), wasting (19.4% vs 8.8% vs 6.9%, *P* < .001), and overweight (20.1% vs 6.4% vs 2.0%, *P* < .001).

**Table 2:** Demographic and Anthropometric Characteristics of Children Aged 6-59 Months, by 3 Selected Data Sources in Ghana

		**GMICS (N = 6532)**	**GDHS (N = 2141)**	**GSPS (N = 1608)**	***P *** **Value**
		**No. (%)**	**No. (%)**	**No. (%)**	
Sex	Male	3317 (50.8)	1071 (50.0)	802 (49.9)	0.723
	Female	3215 (49.2)	1070 (50.0)	806 (50.1)	
Age group (mon)	6-23	2087 (32.0)	791 (36.9)	422 (26.2)	<0.001
	24-59	4445 (68.0)	1350 (63.1)	1186 (73.8)	
Stunting	Yes	1899 (29.1)	643 (30.0)	570 (35.4)	<0.001
	No	4633 (70.9)	1498 (70.0)	1038 (64.6)	
Wasting	Yes	449 (6.9)	188 (8.8)	312 (19.4)	<0.001
	No	6083 (93.1)	1953 (91.2)	1296 (80.6)	
Underweight	Yes	1152 (17.6)	320 (14.9)	400 (24.9)	<0.001
	No	5380 (82.4)	1821 (85.1)	1208 (75.1)	
Overweight	Yes	129 (2.0)	136 (6.4)	324 (20.1)	<0.001
	No	6403 (98.0)	2005 (93.6)	1284 (79.9)	
		**Median (IQR)**	**Median (IQR)**	**Median (IQR)**	
Age (mon)		33.0 (27.0)	30.0 (28.0)	36.0 (26.0)	<0.001
Height (cm)		87.6 (17.3)	86.0 (18.5)	88.0 (17.0)	<0.001
Weight (kg)		11.9 (4.4)	11.7 (4.6)	12.0 (5.0)	<0.001
BMIZ		-0.2 (1.4)	-0.1 (1.6)	0.2 (3.0)	<0.001
WHZ		-0.4 (1.4)	-0.3 (1.6)	0.1 (2.9)	<0.001
HAZ		-1.4 (1.6)	-1.3 (1.9)	-1.3 (2.3)	0.016
WAZ		-1.0 (1.4)	-0.9 (1.4)	-0.7 (2.4)	<0.001

Abbreviations: GMICS, Ghana Multiple Indicator Cluster Survey; GDHS, Ghana Demographic Health Survey; GSPS, Ghana Socioeconomic Panel Survey; IQR, Interquartile range; BMIZ, BMI-for-age; HAZ, height-for-age; WHZ, weight-for-height; WAZ, weight-for-age.

**Figure 1:**
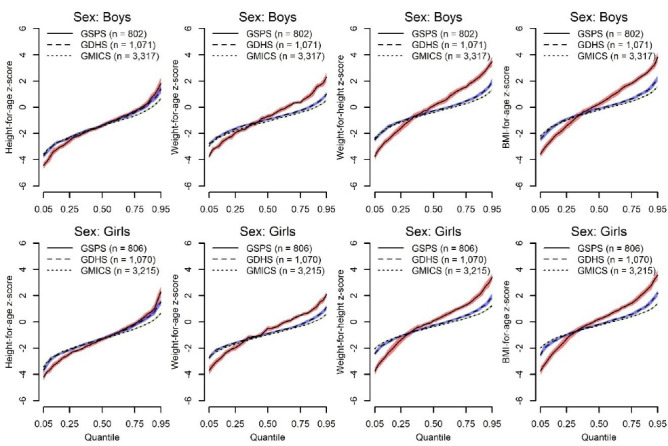
Quantile Regression Intercept Values of Anthropometric z-Scores Comparing 3 Selected Data Sources in Ghana, by Sex. Notes: The lines connect the fit estimates for each quantile, with polygons representing the 95% confidence intervals for the estimates. Non-overlapping confidence polygons indicate significant differences. Abbreviations: GMICS, Ghana Multiple Indicator Cluster Survey; GDHS, Ghana Demographic Health Survey; GSPS, Ghana Socioeconomic Panel Survey.

Figure 1 shows the results of the quantile regression estimates of the anthropometric z-scores. For both boys and girls, the quantile regression lines estimated from the GSPS, GMICS and the GDHS crossed at about the 0.25th quantile for WAZ z-scores, WHZ z-scores, and BMIZ z-scores; with lower estimates for the GSPS below the 0.25th quantile and higher estimates afterwards. For both sexes, the HAZ z-score estimates were similar for the GDHS and GSPS data sources above the 0.25th quantile but lower values were recorded for the GSPS compared the GDHS and GMICS below this quantile. In general, the estimates for the GDHS and GMICS data sources were similar across all quantiles, except for estimates above the 90th quantile where the GMICS had lower values.

**Figure 2:**
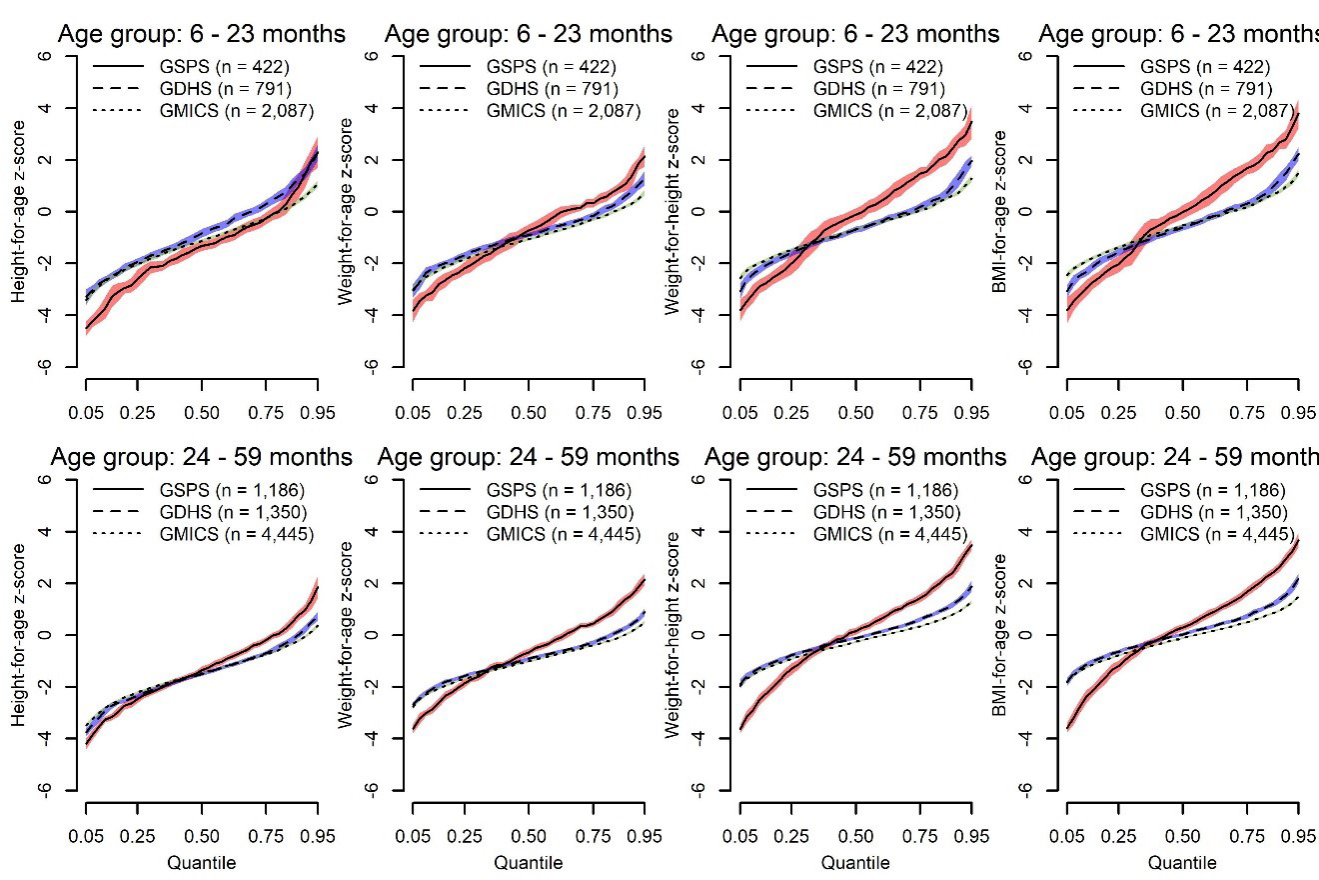
Quantile Regression Intercept Values of Anthropometric z-Scores Comparing 3 Selected Data Sources in Ghana, by Age Group. Notes: The lines connect the fit estimates for each quantile, with polygons representing the 95% confidence intervals for the estimates. Non-overlapping confidence polygons indicate significant differences. Abbreviations: GMICS, Ghana Multiple Indicator Cluster Survey; GDHS, Ghana Demographic Health Survey; GSPS, Ghana Socioeconomic Panel Survey.

 For the comparison of younger (6-23 months) and older (24-59 month) children (Figure 2), the quantile regression lines estimated from the GSPS, GMICS and the GDHS crossed at various quantiles for WAZ z-scores, WHZ z-scores, and BMIZ-for-age z-scores; with lower estimates for the GSPS at the lower quantiles and higher estimates at the higher quantiles. Again, the estimates for the GDHS and GMICS data sources were similar across all quantiles, except for estimates above the 90th quantile where the GMICS had lower values.

 Based on the eCIAF, the overall prevalence estimate of malnutrition at the national level was higher among children in the GSPS (57.3%, 95% CI: 53.9%–60.6%), followed by those in the GDHS (39.7%, 95% CI: 37.0%–42.5%), and then those in the GMICS (31.2%, 95% CI: 29.3%–33.1%) (Table 3). The prevalence estimate of undernutrition ranged from 30.0%, 95% CI: 28.1%–31.8% (in the GMICS) to 45.5%%, 95% CI: 42.0%–49.1% (in the GSPS), while the prevalence estimate of over-nutrition ranged from 2.1%, 95% CI: 1.5%–2.7% (in the GMICS) to 17.8%%, 95% CI: 15.2%–20.4% (in the GSPS).

**Table 3:** Prevalence of Malnutrition Based on the CIAF Among Children Aged 6–59 Months, by 3 Selected Data Sources in Ghana

**Group**	**Anthropometric Status**	**GMICS (N = 6532)**	**GDHS (N = 2141)**	**GSPS (N = 1608)**
		**CIAF - Prevalence (95% CI)**		
A	No failure	70.0 (68.2–71.9)	63.0 (60.2–65.8)	54.5 (50.9–58.1)
B	Wasting only	1.5 (1.1–2.0)	3.2 (2.4–4.0)	4.2 (3.0–5.5)
C	Wasting and underweight	2.4 (1.8–2.9)	3.4 (2.5–4.3)	9.0 (7.2–10.8)
D	Stunting, wasting, and underweight	1.6 (1.2–1.9)	1.7 (1.1–2.3)	5.6 (4.3–7.0)
E	Stunting and underweight	8.4 (7.4–9.4)	8.5 (7.0–10.0)	7.6 (5.9–9.2)
F	Stunting only	14.5 (13.1–15.9)	19.5 (17.3–21.6)	18.4 (15.8–21.0)
Y	Underweight only	1.5 (1.1–1.9)	0.8 (0.4–1.2)	0.8 (0.3–1.2)
B to Y	Overall undernutrition (CIAF)	30.0 (28.1–31.8)	37.0 (34.2–39.9)	45.5 (42.0–49.1)
		**eCIAF - Prevalence (95% CI)**		
A	No failure	68.8 (66.9–70.7)	60.2 (57.5–63.0)	42.7 (39.4–46.1)
B	Wasting only	1.5 (1.1–2.0)	3.2 (2.4–4.0)	4.2 (3.0–5.5)
C	Wasting and underweight	2.4 (1.8–2.9)	3.4 (2.5–4.3)	9.0 (7.2–10.8)
D	Stunting, wasting, and underweight	1.6 (1.2–1.9)	1.7 (1.1–2.3)	5.6 (4.3–7.0)
E	Stunting and underweight	8.4 (7.4–9.4)	8.5 (7.0–10.0)	7.6 (5.9–9.2)
F	Stunting only	13.6 (12.3–15.0)	17.0 (15.1–18.9)	12.3 (10.2–14.4)
Y	Underweight only	1.5 (1.1–1.9)	0.8 (0.4–1.2)	0.8 (0.3–1.2)
G	Stunting and overweight	0.9 (0.5–1.2)	2.5 (1.6–3.4)	6.1 (4.7–7.4)
H	Overweight only	1.2 (0.7–1.8)	2.7 (1.9–3.5)	11.7 (9.5–14.0)
B to H	Overall malnutrition (eCIAF)	31.2 (29.3–33.1)	39.8 (37.0–42.5)	57.3 (53.9–60.6)

Abbreviations: GMICS, Ghana Multiple Indicator Cluster Survey; GDHS, Ghana Demographic Health Survey; GSPS, Ghana Socioeconomic Panel Survey; CIAF, composite index of anthropometric failure; eCIAF, extended composite index of anthropometric failure.

 Comparing prevalence estimates of the malnutrition subtypes in the CIAF between the data sources (Table 3), we found that 4 of the 8 anthropometric failure subtypes (ie, wasting only, stunting only, overweight only, and stunting-and-overweight) were higher in the GDHS compared to the GMICS. Similarly, 5 out of the 8 anthropometric failure subtypes (ie, wasting only, overweight only, wasting-and-underweight, stunting-and-overweight, and stunting-and-wasting-and-overweight) were higher in the GSPS compared to the GMICS. The comparison between the GSPS and GDHS showed that 4 out of the 8 anthropometric failure subtypes (ie, overweight only, wasting-and-underweight, stunting-and-overweight, and stunting-and-wasting-and-overweight) were higher in the GSPS. There were no significant differences between the 3 data sources in the prevalence estimates for the underweight only and stunting-and-underweight failure subtypes.

 Further, we used a two-group CFA, which is a non-parametric statistical approach, to cross-check the differences in the prevalence estimates of the anthropometric failure subtypes identified in Table 3. In general, the two-group CFA confirmed that all the undernutrition subtypes identified in Table 3 as having different prevalence estimates in the 3 data sources had statistically significant differences (Table 4). Further, in instances where there were marginal overlaps between the 95% confidence intervals around prevalence estimates between 2 data sources, the two-group CFA indicated those groups were significantly different. For example, in Table 3, the 95% confidence intervals around the prevalence estimates of stunting only and underweight marginally overlapped between the GSPS and GMICS, however, the two-group CFA indicated that these 2 undernutrition failure subtypes were indeed discrimination types (ie, there were statistically significant differences in the prevalence estimates for these failure subtypes from the 2 data sources) (Table 4 panel C).

**Table 4:** Identifying the Discrimination Subtypes of the CIAF Among Children Aged 6–59 Months, Using a Two-Group CFA

**A: Comparison Between GDHS and GMICS**								
**Configuration**			**Frequencies**				**Significance Tests**	**Discrimination Type**
			**GDHS**		**GMICS**			
**Stunting**	**Wasting**	**Underweight**	**Observed**	**Expected**	**Observed**	**Expected**	***P *** **Value**	
Yes	Yes	Yes	40	50.4	164	153.6	.015	
Yes	Yes	No	0	0.0	0	0.0	1.000	
Yes	No	Yes	188	217.5	693	663.5	.002	True
Yes	No	No	415	359.7	1042	1097.3	.000	True
No	Yes	Yes	74	62.5	179	190.5	.014	
No	Yes	No	74	44.4	106	135.6	.000	True
No	No	Yes	18	33.1	116	100.9	.001	True
No	No	No	1332	1373.5	4232	4190.5	.002	True
**B: Comparison Between GSPS and GDHS**								
**Configuration**			**Frequencies**				**Significance Tests**	**Discrimination Type**
			**GSPS**		**GDHS**			
**Stunting**	**Wasting**	**Underweight**	**Observed**	**Expected**	**Observed**	**Expected**	***P *** **Value**	
Yes	Yes	Yes	103	61.3	40	81.7	.000	True
Yes	Yes	No	0	0.0	0	0.0	1.000	
Yes	No	Yes	135	138.5	188	184.5	.043	
Yes	No	No	332	320.4	415	426.6	.021	
No	Yes	Yes	148	95.2	74	126.8	.000	True
No	Yes	No	61	57.9	74	77.1	.060	
No	No	Yes	14	13.7	18	18.3	.141	
No	No	No	815	920.9	1332	1226.1	.000	True
**C: Comparison between GSPS and GMICS**								
**Configuration**			**Frequencies**				**Significance Tests**	**Discrimination Type**
			**GSPS**		**GMICS**			
**Stunting**	**Wasting**	**Underweight**	**Observed**	**Expected**	**Observed**	**Expected**	***P *** **Value** ^a^	
Yes	Yes	Yes	103	52.7	164	214.3	.000	True
Yes	Yes	No	0	0.0	0	0.0	1.000	
Yes	No	Yes	135	163.6	693	664.4	.001	True
Yes	No	No	332	271.4	1042	1102.6	.000	True
No	Yes	Yes	148	64.6	179	262.4	.000	True
No	Yes	No	61	33.0	106	134.0	.000	True
No	No	Yes	14	25.7	116	104.3	.002	True
No	No	No	815	997.0	4232	4050.0	.000	True

Abbreviations: GMICS, Ghana Multiple Indicator Cluster Survey; GDHS, Ghana Demographic Health Survey; GSPS, Ghana Socioeconomic Panel Survey; CIAF, composite index of anthropometric failure; CFA, configural frequency analysis. ^a^ *P* values were based on Fisher exact test.

**Figure 3:**
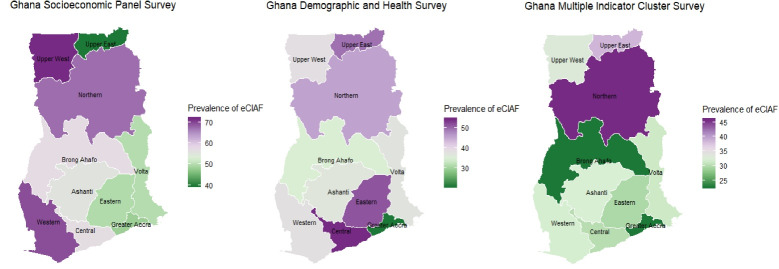
Regional Prevalence Estimates of Overall Malnutrition Based on the eCIAF Among Children Aged 6–59 Months, by 3 Selected Data Sources in Ghana. Abbreviation: eCIAF, extended composite index of anthropometric failure.

 The prevalence of malnutrition calculated at the regional level using the eCIAF is shown in Figure 3. The results from each of the 3 data sources reflect wide variations in regional prevalence of malnutrition in the country. A low prevalence of 40% was estimated for the Upper East region and a high of 72% for the Upper West region in the GSPS, while a low of 21% was estimated for the Greater Accra region and a high of 54% for the Central region in the GDHS. In the GMICS, a low prevalence of 23% was estimated for the Brong Ahafo region and a high of 46% for the Northern region.

 Based on the GDHS, over a 6-year period of 2008-2014, Ghana had a 37% reduction in the prevalence of stunting, 47% reduction in the prevalence of wasting, and 53% reduction in the prevalence of overweight (Table 5). Over a similar 6/7-year period (2011-2017/2018), the GMICS recorded a 40% decrease in the prevalence of stunting, 1% reduction in the prevalence of wasting, and 30% reduction in the prevalence of overweight (Table 5). Whilst the 2014 estimates from the GDHS indicate that the 2025 WHO global nutrition targets were met for both wasting and overweight, the target for wasting was not met in the latest estimates from the 2017-2018 GMICS.

**Table 5:** Ghana’s Progress Towards Achieving the WHO Global Nutrition Targets 2025

**Nutritional Indicator**	**Data Source**				**2025 WHO Global Nutrition Targets **
	**GDHS**		**GMICS**		
	**Baseline**	**Follow-up**	**Baseline**	**Follow-up**	
	**2008**	**2014**	**2011**	**2017-2018**	
			**Prevalence (%)**		
Stunting	30.0	18.8	29.1	17.5	40% reduction by 2025
Wasting	8.8	4.7	6.9	6.8	< 5% prevalence rate
Overweight	6.4	3.0	2.0	1.4	< 6% prevalence rate
			**Change in Prevalence Between Baseline and Follow-up**		
Stunting	Decrease of 37%		Decrease of 40%		
Wasting	Decrease of 47%		Decrease of 1%		
Overweight	Decrease of 53%		Decrease of 30%		

Abbreviations: GMICS, Ghana Multiple Indicator Cluster Survey; GDHS, Ghana Demographic Health Survey; WHO, World Health Organization. Notes: Baseline prevalence estimates are based on the data used in the current study. Follow-up prevalence estimates are taken from the next available survey year. The figures reported for baseline included children 6-59 months, while follow-up figures included children 0-59 months.

## Discussion

 This study sought to estimate the overall prevalence of early childhood malnutrition in Ghana at both national and regional levels, while comparing these estimates across 3 data sources. The results showed that the prevalence estimates for stunting (29.1% to 35.4%), wasting (6.9% to 19.4%), and overweight (6.4% to 20.1%) varied considerably across data sources. Recently, de Onis et al^46^ created 5 prevalence thresholds (ie, very low, low, medium, high, and very high) for describing the severity of malnutrition in a population, with very low and low classifications matching the targets for SDG 2.2. These thresholds are expected to facilitate prioritization and trigger action for targeted interventions. Based on these thresholds, each data source classified the prevalence of early childhood malnutrition differently. For example, whilst the GMICS identifies overweight to be very low in the country, the GDHS and GSPS classifies the country as having medium and very high prevalence levels of overweight, respectively. Although there are no prevalence thresholds available to describe the overall level of childhood malnutrition based on indices such as CIAF and eCIAF, our estimates of eCIAF showed that between nearly one-third (ie, estimates from GMICS) and half (ie, estimates from GSPS) of Ghanaian children aged 6-59 months had at least one form of malnutrition over the study period. Thus, it is likely that the eCIAF estimates from the 3 data sources may inform different prioritization schemes for children requiring urgent interventions. The use of the CIAF and eCIAF allows for distinguishing children who are experiencing single versus multiple anthropometric failures. This distinction is especially important because children with multiple anthropometric failures are more likely than those with only one to experience ill-health and face a higher mortality risk,^14,18^ and thus, should be prioritized for interventions.

 Similar to the national level estimates, our results showed that the prevalence estimates of childhood malnutrition varied across the regions, and among the 3 data sources. For example, although the GDHS estimated higher prevalence rates for the Central, Eastern and Upper East regions, the GSPS estimated lower prevalence rates for the Eastern and Upper East regions, and the GMICS estimated lower prevalence rates for the Central and Eastern regions. Some of the observed variations may be due to the differences in the time periods during which the surveys were undertaken and potentially sampling issues, where the GSPS reported that clusters in the Upper East and Upper West regions were over sampled to allow for a reasonable number of households to be interviewed. It is also possible that the 3 data sources captured different but real variations in early childhood malnutrition in the country. Thus, using multiple data sources as national surveillance tools could provide a broader and more comprehensive assessment of the extent of early childhood malnutrition at sub-national levels, which may be instrumental for prioritizing and designing interventions. With these comprehensive assessments at their disposal, policy-makers could be more empowered to make informed decisions. For example, based on the totality of evidence from the 3 data sources, targeted interventions to mitigate malnutrition, especially in the regions with the highest prevalence rates (ie, Upper West, Central, and Northern), could reduce the level of inequities across the country. One of the key lessons the country has learned in the implementation of the MDGs on childhood malnutrition was the need to decentralize intervention programs development.^10^ To this end, Ghana has since been moving towards decentralized policy-making and program development to tackle early childhood malnutrition from the regional, municipal and district levels.

 Many countries like Ghana whose economies are in transition are experiencing the dual burden of malnutrition,^17^ where there is the co-occurrence of high prevalence of under- and over-nutrition among children. Both under- and over-nutrition can lead to significant co-morbidities which can, if unaddressed, result in mortality. Finding ways to ensure that health systems respond to both of these categories and that health promotion plans are in place for diverse regions and populations is essential for successful interventions to reduce child malnutrition. In our study, the GMICS and GDHS, being the major data sources used to track childhood indicators, revealed low to medium overweight prevalence rates of 2.0% and 6.4%, respectively, while the GSPS showed a much higher rate of 20.1%. Other studies over similar time frames as ours conducted among school-aged children (ie, 5-15 years) mainly in urban sectors have reported overweight prevalence rates in excess of 10%; including overweight prevalence of 26.7% reported by Mahammed and Vuvor,^47^ 18.1% reported by Aryeetey et al,^48^ and 16.4% reported by Adom et al.^49^ Although the overweight prevalence rates reported in these studies were conducted in slightly older children and mainly in urban settings, it is worth monitoring the overweight prevalence rates in the 0-59 months group to avert the possibility of the country being taken by surprise with the double burden of malnutrition in this age group. This is particularly important given that the estimates from the GSPS are hinting the possibility of a co-occurrence of under- and over-nutrition leading to a double burden of malnutrition in the country.

 As part of efforts to meet the United Nations’ SDGs of ending all forms of malnutrition by 2030, the World Health Assembly Resolution 65.6 endorsed a comprehensive implementation plan on maternal, infant and young child nutrition in 2012, which specified a set of 6 global nutrition targets that should be met by 2025. These include a 40% reduction in stunting among children under 5 years, reduction of prevalence of childhood wasting to less than 5%, and reduction of prevalence of childhood overweight to less than 6%.^20^ To provide some context for Ghana’s progress towards meeting the 2025 targets, we compared the latest prevalence estimates from the 2014 GDHS and the 2017-2018 GMICS with our estimates (Table 5). Using the GMICS data source, we found that the targets for stunting and overweight have been met whilst the target for wasting is yet to be met. On the other hand, using the GDHS, we found that the targets for wasting and overweight have been achieved whilst the target for stunting is yet to be achieved. Some of these achievements can be attributed to the implementation of several nutrition-sensitive interventions to curb malnutrition among different vulnerable groups, such as pregnant women, postpartum women, children under 5 years, and primary school children. Examples of these interventions include free antenatal care services, iron and folate supplementation for pregnant women, and school feeding programs.^23^

 Globally, one of the main challenges of meeting the targets set for the SDGs has been the lack of comprehensive data to enable consistent monitoring of progress.^19^ In addition to providing overall prevalence estimates, our study also sought to explore how comparable 3 data sources (ie, GMICS, GDHS, and GSPS) were to one another; and in turn provided an assessment of how their complementary usage (ie, triangulation) could serve as national surveillance tools to monitor childhood malnutrition in the country. We found that the GSPS prevalence estimates of malnutrition were consistently higher than the estimates from the GDHS and GMICS. To better understand why that was the case, we plotted the entire distributions of the anthropometric z-scores using quantile regressions. These plots compared estimates between the GSPS, GDHS, and GMICS separately for boys and girls, as well as for the younger (6-23 months) and older (24-59 month) children (Figures 1 and 2). For all groups, compared to the GDHS and GMICS, almost all the anthropometric z-scores were consistently lower in the GSPS below the 0.25th quantile and higher afterwards. This explains why although the median BMIZ, WHZ, HAZ, and WAZ were higher in the GSPS compared with the GDHS and GMICS, the proportions of children whose z-scores were <-2.0 were higher in the GSPS. Consequently, the GSPS also provided higher estimates of childhood over-nutrition compared to the GDHS and GMICS. These important differences are worth exploring further in the subsequent survey cycles.

###  Strengths and Limitations

 A major strength of the study is the use of nationally representative data sets, which provided robust prevalence estimates of early childhood malnutrition. Although some of the data for child age may be based on mother’s or caretaker’s reports, the anthropometric indicators (ie, height and weight) were objectively measured by well-trained technicians,^26-28^ reducing possible misclassification of early childhood malnutrition. In our attempt to compare how multiple data sources align with their estimates of early childhood malnutrition in Ghana, we selected 3 nationally representative surveys closest in time to one another (ie, 2008 GDHS, 2009-2010 GSPS, and 2011 GMICS). Yet still, 2 of the data sources were 3 years apart, and the results of the study should be interpreted with this consideration. However, we did not find any clear trend in the prevalence estimates based on the year in which the surveys were conducted. Overall, malnutrition prevalence estimates were higher in 2009-2010 GSPS than estimates in the 2008 GDHS and 2011 GMICS.

## Conclusion

 To the best of our knowledge, this study is the first to reveal the variations in the overall prevalence estimates of early childhood malnutrition using indices such as the CIAF and eCIAF at the national and regional levels in Ghana across 3 nationally representative data sources. Depending on the data source adopted, our estimates of eCIAF showed that between one-third and half of all Ghanaian children aged 6-59 months had at least one form of malnutrition over the study period. These estimates are quite alarming and should play a complementary role to the usually reported measures of stunting, wasting, and overweight when interpreting the level and severity of malnutrition in the country to inform policy decisions. We have also found regions with the highest prevalence rates to guide policy-makers design targeted interventions. By comparing estimates across the 3 data sources, our study has demonstrated that triangulating multiple data sources as national surveillance tools to monitor early childhood malnutrition would enrich the evidence base for effective policy-making. In designing effective interventions, policy-makers need estimates of both incidence and prevalence of malnutrition. Although the MICS and DHS are popular data sources in Ghana and around the world for estimating prevalence of malnutrition, the GSPS being a panel survey provides a promising opportunity to measure incidence of malnutrition at a nationally representative level; an indicator virtually non-existent in the developing world.

## Acknowledgements

 We are grateful to all the families who participated in the 2008 GDHS, 2009-10 GSPS, and 2011 GMICS surveys. Also, we are grateful to the DHS and MICS programs for kindly providing access to their respective datasets. Lastly, the 2009-10 GSPS data used in this study is a joint effort undertaken by the Institute of Statistical, Social and Economic Research (ISSER) at the University of Ghana, and the Economic Growth Centre (EGC) at Yale University. At the same time, ISSER and the EGC are not responsible for the estimations reported by the authors.

## Ethical issues

 This study is based on data from 3 surveys which fulfilled all ethical requirements and participants’ records were anonymized in the respective databases, fully concealing the identities of all participants. Since the data are publicly available, no further approvals are required for the retrospective use of these data.

## Competing interests

 Authors declare that they have no competing interests.

## Authors’ contributions

 JPK led the conceptualization of the study, design and acquisition of the data, statistical analysis, drafting the manuscript, and interpretation of the results. JA, TM, EJC, ED, and AN supported drafting of the manuscript, data analysis, and critically reviewed the manuscript.

## References

[1] Zheng JS, Liu H, Li J (2014). Exclusive breastfeeding is inversely associated with risk of childhood overweight in a large Chinese cohort. J Nutr.

[2] Wang Y, Lim H (2012). The global childhood obesity epidemic and the association between socio-economic status and childhood obesity. Int Rev Psychiatry.

[3] Pomati M, Nandy S (2019). Assessing Progress towards SDG2: Trends and Patterns of Multiple Malnutrition in Young Children under 5 in West and Central Africa. Child Indic Res.

[4] Black RE, Victora CG, Walker SP (2013). Maternal and child undernutrition and overweight in low-income and middle-income countries. Lancet.

[5] Glewwe P, Miguel EA (2017). 2007.

[6] Dewey KG, Begum K (2011). Long-term consequences of stunting in early life. Matern Child Nutr.

[7] Swinburn BA, Kraak VI, Allender S (2019). The Global Syndemic of Obesity, Undernutrition, and Climate Change: The Lancet Commission report. Lancet.

[8] Martins VJB, Florê TMMT, Santos CDL, Vieira MDFA, Sawaya AL (2011). Long-Lasting Effects of Undernutrition. Int J Environ Res Public Health.

[9] The United Nations Children’s Fund (UNICEF). Levels and trends in child malnutrition: key findings of the 2019 Edition of the Joint Child Malnutrition Estimates. https://www.who.int/nutgrowthdb/jme-2019-key-findings.pdf?ua=1. Accessed March 7, 2020. Published 2019.

[10] UNDP. Ghana Millenium Development Goals. https://www.gh.undp.org/content/ghana/en/home/library/poverty/2015-ghana-millennium-development-goals-report.html. Accessed July 10, 2020. Published 2015.

[11] de Groot R, Palermo T, Handa S, Ragno LP, Peterman A (2017). Cash Transfers and Child Nutrition: Pathways and Impacts. Dev Policy Rev.

[12] Ziba M, Kalimbira AA, Kalumikiza Z (2018). Estimated burden of aggregate anthropometric failure among Malawian children. South African J Clin Nutr.

[13] Pei L, Ren L, Yan H (2014). A survey of undernutrition in children under three years of age in rural Western China. BMC Public Health.

[14] Svedberg P. Poverty and Undernutrition: Theory, Measurement and Policy. New Delhi: Oxford India Paperbacks; 2005.

[15] Nandy S, Irving M, Gordon D, Subramanian SV, Smith GD (2005). Poverty, child undernutrition and morbidity: New evidence from India. Bull World Health Organ.

[16] Kuiti BK, Bose K (2018). The Concept of Composite Index of Anthropometric Failure (CIAF): Revisited and Revised. Anthropol - Open J.

[17] Bejarano IF, Oyhenart EE, Torres MF (2019). Extended composite index of anthropometric failure in Argentinean preschool and school children. Public Health Nutr.

[18] Goswami M (2016). Prevalence of under-nutrition measured by composite index of anthropometric failure (CIAF) among the Bhumij children of northern Odisha, India. J Nepal Paediatr Soc.

[19] Kumar S, Kumar N, Vivekadhish S (2016). Millennium development goals (MDGS) to sustainable development goals (SDGS): Addressing unfinished agenda and strengthening sustainable development and partnership. Indian J Community Med.

[20] World Health Organization. Global Nutrition Targets 2025. https://www.who.int/nutrition/global-target-2025/discussion-paper-extension-targets-2030.pdf?ua=1. Accessed March 23, 2020. Published 2014.

[21] Hure A, Oldmeadow C, Attia J (2016). Invited Commentary: Improving Estimates of Severe Acute Malnutrition Requires More Data. AJE.

[22] Sipsma H, Ofori-Atta A, Canavan M, Osei-Akoto I, Udry C, Bradley EH (2013). Poor mental health in Ghana: Who is at risk?. BMC Public Health.

[23] Boah M, Azupogo F, Amporfro DA, Abada LA (2019). The epidemiology of undernutrition and its determinants in children under five years in Ghana. PLoS Med.

[24] Tette EMA, Sifah EK, Nartey ET (2015). Factors affecting malnutrition in children and the uptake of interventions to prevent the condition. BMC Pediatr.

[25] Glover-Amengor M, Agbemafle I, Hagan LL (2016). Nutritional status of children 0-59 months in selected intervention communities in northern Ghana from the africa RISING project in 2012. Arch Public Health.

[26] Ghana Statistical Service. Ghana Demographic and Health Survey 2008. https://www.dhsprogram.com/pubs/pdf/FR221/FR221%5B13Aug2012%5D.pdf. Accessed March 23, 2020. Published 2008.

[27] Aryeetey E, Osei-akoto I, Osei RD, Udry C. Ghana Socioeconomic Panel Survey - Report of the Baseline Survey. https://catalog.ihsn.org/index.php/citations/60394. Accessed April 3, 2020. Published 2011.

[28] Ghana Health Service. Ghana Multiple Indicator Cluster Survey with an Enhanced Malaria Module and Biomarker. https://www.dhsprogram.com/publications/publication-FR262-Other-Final-Reports.cfm. Accessed April 3, 2020. Published 2011.

[29] Foster BA, Farragher J, Parker P, Sosa ET (2015). Treatment Interventions for Early Childhood Obesity: A Systematic Review. Acad Pediatr.

[30] Tchoubi S, Sobngwi-Tambekou J, Noubiap JJN, Asangbeh SL, Nkoum BA, Sobngwi E (2015). Prevalence and risk factors of overweight and obesity among children aged 6-59 months in Cameroon: A multistage, stratified cluster sampling nationwide survey. PLoS One.

[31] Myatt M, Khara T, Dolan C, Garenne M, Briend A (2019). Improving screening for malnourished children at high risk of death: A study of children aged 6-59 months in rural Senegal. Public Health Nutr.

[32] World Health Organization. Recommendations for Data Collection, Analysis and Reporting on Anthropometric Indicators in Children under 5 Years Old. https://www.who.int/nutrition/publications/anthropometry-data-quality-report/en/. Accessed April 3, 2020. Published 2019.

[33] World Health Organization. WHO child growth standards and the identification of severe acute malnutrition in infants and children. http://apps.who.int/iris/bitstream/10665/44129/1/9789241598163_eng.pdf?ua=1. Accessed April 8, 2020. Published 2006.

[34] Koenker R, Hallock KF (2001). Quantile Regression. J Econ Perspect.

[35] Siers SR, Savidge JA, Reed RN (2017). Quantile regression of microgeographic variation in population characteristics of an invasive vertebrate predator. PLoS One.

[36] Stemmler M, Heine JH (2017). Using Configural Frequency Analysis as a Person-centered Analytic Approach with Categorical Data. Int J Behav Dev.

[37] Ghana Statistical Service. Ghana demographic health survey 2014. https://dhsprogram.com/publications/publication-FR307-DHS-Final-Reports.cfm. Accessed April 8, 2020. Published 2014.

[38] Kennedy AN, Kennedy MN. Forestmodel. R package version 0.6 2; 2018.

[39] Dowle M, Srinivasan A, et al. Data.table: Extension of ‘data.frame’. R package version 1.11 4; 2018.

[40] Heine JH, Alexandrowicz RW, Stemmler M. Confreq: Configural Frequencies Analysis Using Log-Linear Modeling. R package version 1.5.4- 3; 2019.

[41] Koenker R. quantreg: Quantile Regression. R package version 5 36; 2018.

[42] Kahle D, Wichham H, Jackson S, Korpela M (2013). ggmap: Spatial Visualization with ggplot2. The R Journal.

[43] Lumley T. Survey: analysis of complex survey samples. R package version 3 32; 2017.

[44] Myatt M, Guevarra E. z scorer: Child Anthropometry z-Score Calculator. R package version 0.3 1; 2019.

[45] Wickham H. ggplot2: Elegant Graphics for Data Analysis. New York: Springer-Verlag; 2016.

[46] De Onis M, Borghi E, Arimond M (2019). Prevalence thresholds for wasting, overweight and stunting in children under 5 years. Public Health Nutr.

[47] Mohammed H, Vuvor F (2012). Prevalence of childhood overweight/obesity in basic school in Accra. Ghana Med J.

[48] Aryeetey R, Lartey A, Marquis GS, Nti H, Colecraft E, Brown P (2017). Prevalence and predictors of overweight and obesity among school-aged children in urban Ghana. BMC Obes.

[49] Adom T, De Villiers A, Puoane T, Kengne AP (2019). Prevalence and correlates of overweight and obesity among school children in an urban district in Ghana. BMC Obes.

